# 4DCT image artifact detection using deep learning

**DOI:** 10.1002/mp.17513

**Published:** 2024-11-14

**Authors:** Joshua W. Carrizales, Mattison J. Flakus, Dallin Fairbourn, Wei Shao, Sarah E. Gerard, John E. Bayouth, Gary E. Christensen, Joseph M. Reinhardt

**Affiliations:** ^1^ Biomedical Engineering University of Iowa Iowa City Iowa USA; ^2^ Medical Physics University of Wisconsin ‐ Madison Madison Wisconsin USA; ^3^ Biological Engineering Utah State University Logan Utah USA; ^4^ Medicine University of Florida Gainesville Florida USA; ^5^ Radiation Medicine Oregon Health and Science University Portland Oregon USA; ^6^ Electrical and Computer Engineering University of Iowa Iowa City Iowa USA

**Keywords:** deep learning, 4DCT, motion artifacts

## Abstract

**Background:**

Four‐dimensional computed tomography (4DCT) is an es sential tool in radiation therapy. However, the 4D acquisition process may cause motion artifacts which can obscure anatomy and distort functional measurements from CT scans.

**Purpose:**

We describe a deep learning algorithm to identify the location of artifacts within 4DCT images. Our method is flexible enough to handle different types of artifacts, including duplication, misalignment, truncation, and interpolation.

**Methods:**

We trained and validated a U‐net convolutional neural network artifact detection model on more than 23 000 coronal slices extracted from 98 4DCT scans. The receiver operating characteristic (ROC) curve and precision‐recall curve were used to evaluate the model's performance at identifying artifacts compared to a manually identified ground truth. The model was adjusted so that the sensitivity in identifying artifacts was equivalent to that of a human observer, as measured by computing the average ratio of artifact volume to lung volume in a given scan.

**Results:**

The model achieved a sensitivity, specificity, and precision of 0.78, 0.99, and 0.58, respectively. The ROC area‐under‐the‐curve (AUC) was 0.99 and the precision‐recall AUC was 0.73. Our model sensitivity is 8% higher than previously reported state‐of‐the‐art artifact detection methods.

**Conclusions:**

The model developed in this study is versatile, designed to handle duplication, misalignment, truncation, and interpolation artifacts within a single image, unlike earlier models that were designed for a single artifact type.

## 1 INTRODUCTION

Lung cancer is one of the most common forms of cancer, behind only prostate for men and breast cancer for women.^1^ However, it remains the deadliest of all forms of cancer in the United States of America.^2^, ^3^, ^4^ Current treatment plans include chemotherapy, surgery, immunotherapy, and radiation.^4^ Of these treatment options, advances in radiation therapy (RT) over the past two decades have increased survival rates and patient quality of life.^5^, ^6^, ^7^ Four‐dimensional computed tomography^8^, ^9^ (4DCT) is a valuable tool in RT with 93% of clinicians using it to treat thoracic and abdominal cancer patients.^10^ Its effectiveness lies in facilitating the precise quantification of both tumor movement and respiratory motion. A 4DCT^11^ scan consists of multiple 3DCT scans obtained at different breathing phases throughout a patient's respiratory cycle. Each 3D scan is comprised of multiple image stacks^12^ of axial slices.^12^ These stacks are combined to form a cohesive 3D volume,^13^ creating an image volume that covers the entire patient's lung anatomy. Several groups^14^, ^15^, ^16^, ^17^, ^18^ have investigated methods to use 4DCT to improve lung cancer treatments through functional avoidance RT. The study conducted in this paper utilizes a treatment simulation 4DCT for functional avoidance RT which provides a significant advantage by pinpointing areas of high functioning lung tissue. This information guides the treatment planning process, allowing for the precise sparing of these regions to potentially minimize post‐treatment toxicities. Unfortunately, 4DCT scans rely heavily on consistent patient breathing patterns and are therefore susceptible to image artifacts that limit their clinical applicability.^8^, ^12^, ^19^, ^20^, ^21^, ^22^, ^23^, ^24^

**FIGURE 1:**
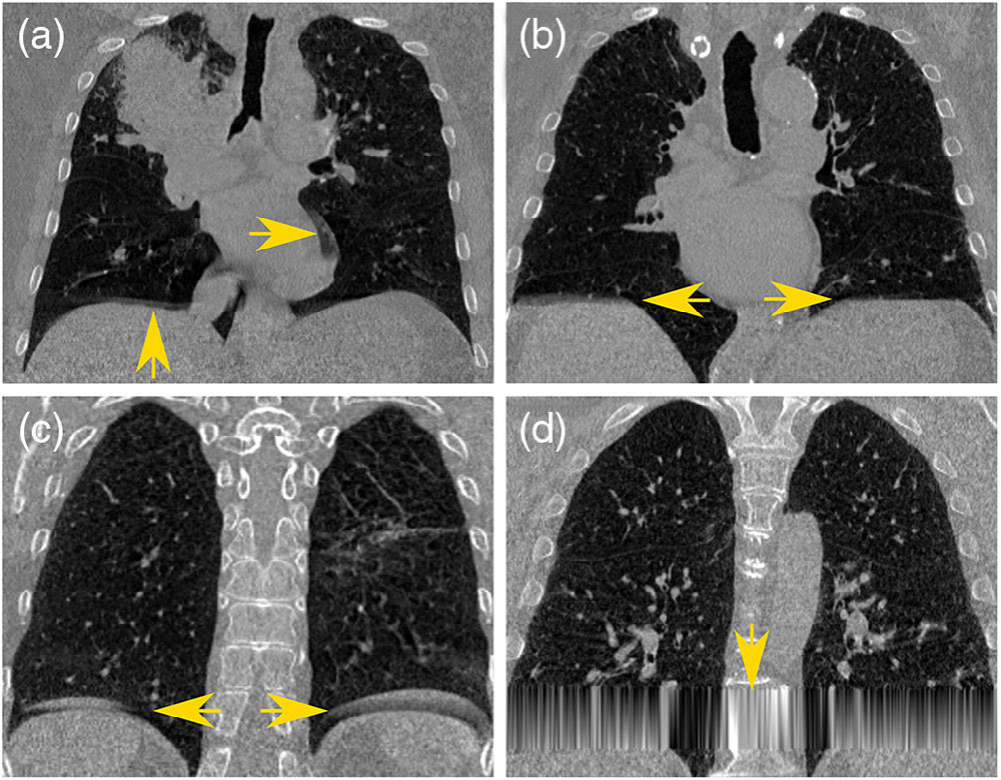
(a) Misalignment artifact characterized by a distinct discontinuity in the left lung and a truncation artifact in the right lung. (b) Truncation artifact is seen as a flattened diaphragm. (c) Duplication artifact marked by a double diaphragm. (d) Interpolation artifact which is a result of missing data in the image and a simple interpolation to fill in the missing data.

A full consensus on how to define and identify types of artifact has not been established. Additionally, previous artifact detection algorithms are limited to a single artifact type only.^25^, ^26^, ^27^, ^28^ Artifacts are in part caused by irregular patient breathing patterns, anatomy motion in the same or opposite direction as the scanner, and 4DCT binning methods. As a result, many groups use their own definitions for how artifacts are to be defined.^12^, ^24^, ^25^, ^26^, ^28^ This inconsistency has resulted in multiple names and definitions for the same type of artifact and even the same name or definition for different artifacts.^12^, ^24^ In this work, we define four types of artifacts: misalignment, truncation, duplication, and interpolation (Figure 1).

Misalignment artifacts (Figure 1a) have previously been called double structure heart, duplicate, or left undefined.^21^, ^29^ We define a misalignment artifact to be an image stack where there is a disparity in anatomical positioning due to heart motion. These artifacts are primarily found in the left lung near the heart and cause a portion of the lung to appear misaligned in comparison to the rest of the lung structure.

Figure 1b shows truncation artifacts, also known as gap artifacts.^12^, ^24^, ^25^ These artifacts manifest as a discrepancy in lung volume between neighboring stacks, typically occurring when a regular breath is succeeded by a shallow breath. As a result, the anatomical structures seem truncated, particularly noticeable near the diaphragm, which may appear flattened.

Duplication artifacts (Figure 1c), also referred to as double structure or overlap,^12^, ^24^, ^25^, ^27^ arise from irregular breathing patterns wherein a regular breath is succeeded by a deep inhalation, resulting in the duplication of anatomical structures. These artifacts are frequently observed near the diaphragm.

Figure 1d shows interpolation artifacts which have been called anatomical loss, blurring, or missing data by other researchers.^12^, ^27^, ^29^ Interpolation artifacts stem from instances where patients breathe too slowly, cough, or inadvertently pause breathing during continuous table motion, such as helical acquisition. This leads to a noticeable segment of absent data in the acquired images. Some scanner manufacturers (e.g., Siemens) address this issue by performing a linear interpolation from cranial to caudal between adjacent stacks on either side of the missing region. Interpolation artifacts can occur anywhere in the image and will span the entire width of the image laterally.

Many analytical methods of artifact detection have been proposed and used to identify motion artifacts in 4DCT images. Castillo et al. utilized the respiratory trace as a surrogate for finding phases that contain artifacts.^28^ If the respiratory trace was outside acceptable boundaries, then it was marked as “irregular”. This method works well on a broad sense as it is clinical utility is apparent. This allows clinicians to rescan the patient and mitigate artifacts during the scanning procedure. Another popular method for detecting artifacts is to compare adjacent axial slices for substantial differences. Werner et al. compared the utility of mean squared intensity difference (MSD) to using a registration approach where they registered a phantom's motion to patient data and compared this to the original image containing artifacts.^30^ Similarly, Li et al. used deformable image registration to map a breath hold CT scan to the 4DCT phases.^25^ The result can be compared to the original 4DCT phase images and the difference of the two revealed motion artifacts. Another remarkable contribution to artifact detection was the global Fourier analysis work done by Wei et al.^31^ They analyzed the spectral domain of 4DCT phase images and developed a metric that relies on set spectral bands as thresholds for defining the artifact region.^31^ Any frequencies within this region should be considered as artifacts. Moreover, they were able to determine the severity of the artifacts based on the magnitude of their derived metric. Finally, Han et al. utilized normalize cross correlation (NCC) to identify artifacts between adjacent stacks of slices.^12^ They were able to identify artifacts at the stack boundaries and relate a small NCC value to severe artifacts. While these analytical methods have shown great utility in their respective domains, many of them suffer from issues with computing time, processing power, minimal validation subjects, and a narrowed focus on specific types of artifacts. Analytical methods fail to view artifacts as human observers view them. There is a definite need for computer vision and deep learning methods to further advance artifact detection techniques.

Many advancements have been made in improving 4DCT images by using deep learning artifact detection and correction techniques.^27^, ^29^, ^32^ However, most of these advancements have only focused on one or two types of artifacts. There is a need for an artifact detection algorithm that is able to detect multiple types of artifacts and provide information on where an artifact is located. In this work, we developed a 2D deep‐learning model, referred to as AD2, to automatically detect multiple types of respiratory motion artifacts in 4DCT images.

## 2 METHODS

### 2.1 4DCT Acquisition and reconstruction

The 4DCT protocol used in this study was approved by the University of Wisconsin – Madison IRB (NCT02843568). 4DCT images were acquired for 79 non‐small cell lung cancer (NSCLC) patients on a Siemens SOMATOM Definition Edge CT scanner (Siemens Healthineers, Erlangen, Germany) at the University of Wisconsin ‐ Madison with scan parameters of 120 kV, 100 mAs/rotation, 0.5 s tube rotation period, a pitch of 0.09, 76.8 mm beam collimation and 128 detector rows. Images were reconstructed with 512mm field‐of‐view, 1 mm slice thickness, and a medium smooth kernel. Audio guidance breathing instructions were played at 15 beats per minute (BPM) during scan acquisition to encourage consistent breathing.^33^ Informed consent was received from all participants in this study. Additionally, we used publicly available data from the DIR‐Lab at Emory University^34^, ^35^ to check if our model developed works on data acquired at another institution. The DIR‐Lab data was composed of 10 patients with a voxel spacing ranging between [0.9 7 to 1.16 mm] × [0.97 to 1.16 mm] × 2.50 mm. The image dimensions were [256,512] × [256,512] × [94–136]. They utilized a General Electric Discovery ST PET/CT^34^, ^35^ scanner and only acquired data for the inspiration side of the respiratory trace.

The 4DCT scans used in this study are composed of multiple 3D breathing phase volumes, where a breathing phase is dependent on the lung volume amplitude during inspiration (IN) and expiration (EX).^12^ In this work, each 4DCT is reconstructed into 10‐3D volumes denoted 0EX, 20IN, 40IN, 60IN, 80IN, 100IN, 80EX, 60EX, 40EX, and 20EX where 100IN is 100% inspiration and 0EX is exhale.^12^, ^29^ Each phase is a 3DCT image with approximately 10 stacks of data acquired from different respiratory cycles occurring at a target breathing phase.^12^ An image stack refers to multiple consecutive transverse image slices in a 3D breathing phase volume. Each stack of image slices is binned based on the patient's chest amplitude as measured by an external surrogate.^12^ Due to the nature of 4DCT acquisition and reconstruction, images are prone to respiratory motion artifacts caused by irregular patient breathing. Lung cancer patients often have difficulty controlling their depth of inspiration and maintaining a constant breathing frequency.^8^, ^12^, ^19^, ^20^, ^21^, ^25^, ^36^, ^37^ As a result, artifacts occur and may reduce treatment precision, image quality, and may perturb measures used to guide functional avoidance RT.^5^, ^15^, ^38^, ^39^, ^40^, ^41^, ^42^, ^43^, ^44^

### 2.2 Data pre‐processing and augmentation

The 2D artifact detection model, AD2, was trained and validated using data obtained from 28 subjects. Among these subjects, 98‐3D scans containing at least one visible artifact were identified. These scans encompassed both pre and post‐RT treatment stages and were captured across all breathing phases. The dataset showcased diverse lung appearances, lung volumes, and encompassed multiple artifact types. Notably, the dataset demonstrated a non‐uniform distribution of artifact types; it contained 278 duplication, 122 misalignment, 116 truncation, and 11 interpolation artifacts. All artifacts were identified by a single observer (J.W.C.) as described below. Given the unbalanced distribution of artifacts within the dataset, additional artificial interpolation artifacts were generated to augment the original dataset. This augmentation process aimed to address the imbalance in artifact representation, ensuring a more comprehensive and balanced dataset for training and validation purposes.

**FIGURE 2:**
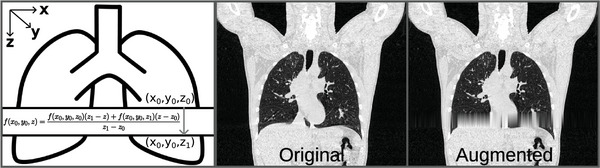
Artificial interpolation artifacts are created using linear interpolation. Stacks of data are removed from an image. Points from the top and bottom of the stack are linearly interpolated to generate the artifact. (Left) Mathematical equation and visual aid for applying the artificial artifact. (Middle) Original CT image. (Right) The same CT image with an artificial artifact.

To generate artificial interpolation artifacts, 75 scans were randomly selected for augmentation. Coronal slices were extracted from each CT scan. In each coronal slice, a random amount of axial slices ranging from 3 to 45 axial slices were removed from the coronal image. Then linear interpolation (Equation 1) was applied to each sagittal column of pixels in the region of missing data (Figure 2):

(1) $$f\left(x_{0},y_{0},z\right)=\frac{f\left(x_{0},y_{0},z_{0}\right)\left(z_{1}-z\right)+f\left(x_{0},y_{0},z_{1}\right)\left(z-z_{0}\right)}{z_{1}-z_{0}},$$

where f(x,y,z) is the Hounsfield unit (HU) intensity and (x,y,z) are positional coordinates. The height of the synthetic interpolation artifacts was randomly generated from a uniform distribution with range 3 to 45 pixels. To assess the realism of our artificial interpolation artifacts, we created a synthetic artifact at the same location as a real interpolation artifact. The absolute difference between the synthetic and real interpolation artifacts did not exceed 2 HU.

A single observer analyzed every 2D coronal slice from each 3D volume for artifacts and established the ground truth (GT) for artifact segmentation. The observer only segmented misalignment, truncation, duplicate, or interpolation artifacts. Artifacts were visually identified by the human observer and segmented at the diaphragm, heart, chest wall boundary, and throughout the lung tissue. An artifact was segmented if it was visible to the human eye.

### 2.3 Deep learning models

#### 2.3.1 U‐Net

**FIGURE 3:**
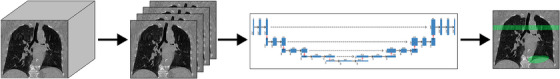
2D slices containing the lungs were extracted from a 3D volume. The 2D slices of size 256 × 256 are passed into the U‐Net model where features are extracted. The model learns what artifacts look like and where they are commonly located. The output is a probability map which can be thresholded to generate a binary mask seen in green.

**FIGURE 4:**
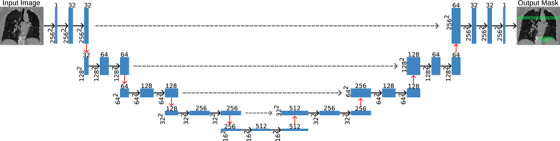
A U‐Net with four encoding layers and four decoding layers was trained. A 2D coronal slice of size 256 × 256 is input and a 2D probability map of where artifacts are located of size 256 × 256 is output. Black arrows are convolutional layers, red arrows are pooling layers, and dashed arrows are skip connections.

In this work, we propose a deep learning approach for direct segmentation of respiratory motion artifacts from 2D slices taken from a 4DCT scan (Figure 3). The goal is to train a model that is able to map input CT images to an output probability map that indicates the likelihood that an artifact is present at a given location. The network input is a single channel consisting of 2D coronal slices of size 256 × 256 pixels. Figure 4 shows the network encoder consisting of four blocks, each containing two convolutional layers with rectified linear unit (ReLU) activation functions. These convolutional layers learn to extract hierarchical features from the input image. Max‐pooling layers are used to reduce the spatial dimensions of the feature maps, effectively capturing increasingly abstract representations of the input image. Dropout layers are applied after each max‐pooling layer to prevent overfitting. After several encoding blocks, the network reaches a bottleneck layer. This layer further extracts high‐level features and acts as a bridge between the encoding and decoding parts of the network. The decoding path is symmetric to the encoding path. It involves up‐sampling the feature maps using transposed convolutional layers to gradually increase the spatial resolution. Concatenation is performed with feature maps from the corresponding encoding block which act as skip connections, allowing the network to preserve high‐resolution features. The final output layer consists of a single convolutional layer with a sigmoid activation function to produce an artifact probability map with the same dimensions as the input image. A balanced cross‐entropy loss^45^ was used to account for the imbalance between the number of voxels labeled artifact versus background. Moreover, the U‐Net^46^ architecture was chosen in this work because of its success in other applications, demonstrating reliability and robustness.^47^, ^48^, ^49^ Additionally, work done by co‐author Shao et al. on 4DCT artifact correction^29^ included a small section devoted to artifact detection where they demonstrated that a U‐Net is well suited to perform artifact detection. This is in contrast to work done by Madesta et al.^27^ where they utilized a fully convolutional neural network to perform artifact detection on artificially generated data. A fully convolutional neural network does not contain skip connections^50^ which are vital to a U‐Net. Skip connections provide important context^46^ for a model's prediction because images may contain different artifacts and different anatomical structures. While we acknowledge the push to newer deep learning architectures such as transformer models,^51^, ^52^ diffusion models,^53^, ^54^, ^55^ and state space models,^56^, ^57^ the purpose of this work was to develop a reliable and easily implemented tool for artifact detection.

#### 2.3.2 Training and validation

The data used for training and validation came from 28 subjects resulting in a comprehensive dataset of 98‐3D volumes containing artifacts. The composition of the training and validation datasets can be found in Table 1. Intensity normalization was applied to each image to scale the intensity values to the range from zero to one. Then the images were resized to 256 × 256 using bilinear interpolation with the Python library OpenCV.^58^ The resulting train/validation split was 0.78/0.22.

**TABLE 1:** Overview of training and validation datasets.

Type of data	Train	Validation
3D volumes	77	21
Total 2D slices	17920	5194
Natural artifact 2D slices	12920	3740
Artificial artifact 2D slices	5000	1454

Figure 3 shows the training pipeline. A 2D coronal slice is input to the U‐net which then produces an artifact probability map. This is then compared to the GT image and the loss function is evaluated to produce a floating point number that informs the model how close it is to the GT. The network weights are adjusted and the training process repeats. The model was trained for 100 epochs using a batch size of 32, a learning rate of 0.00003, and the Adam optimizer.^59^

### 2.4 Artifact probability threshold

**FIGURE 5:**
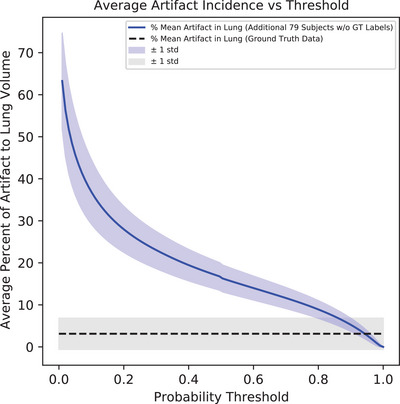
A threshold of 0.94 was found to match the sensitivity of AD2 to the human observer. The volume of artifacts in the lung was calculated as a percentage of the total lung volume for each subject. This metric was calculated at every threshold value to compare to the mean artifact volume in the lung for the GT data. The GT data is not dependent upon the threshold values. Standard deviations are shown as the opaque regions. GT, ground truth.

The AD2 model generates a probability map predicting artifact locations in an image. A probability threshold must be selected to turn the floating point probability map into a binary artifact mask. This threshold is based on a comparison with human observer GT segmentations, ensuring that any artifact visible to a human is also visible to the model. The ratio of artifact volume to lung volume was calculated for the GT dataset and for the model's prediction on an additional 79 scans without GT labels. These additional scans were taken from the rest of the patient population to try and characterize the rest of our data that did not contain GT labels. The ratio was calculated for the model's prediction at various thresholds to find where the model's ratio matched the GT. The selected threshold was identified where the model and GT data showed equal artifact volumes within the lung, which occurred at a threshold value of 0.94, as shown in Figure 5. The model was also tested using thresholds determined by Youden's J statistic^60^ and the F1‐score.^61^

### 2.5 Model evaluation

The model developed in this work was trained on 2D coronal slices from 4DCT scans. However, the model was evaluated on 3D volumes by passing each coronal slice of a 3D CT scan through the model, thresholding the model to produce a binary mask, and concatenating each slice to reconstruct a 3D artifact mask. This method was chosen to simulate the method in which a human observer would segment artifacts in a 3D volume. The receiver operating characteristic (ROC)^60^ curve was utilized to assess the performance of the AD2 model. The ROC curves provided a comprehensive evaluation of the model's ability to discriminate between artifact‐corrupted and artifact‐free voxels. The ROC curve compares the true positive rate (TPR) versus the false positive rate (FPR) at various thresholds where the TPR represents the proportion of pixels correctly identified as artifacts and the FPR quantifies the proportion of pixels incorrectly identified as artifacts. By taking the area under the ROC curve (AUC), we obtain an aggregate measure of the model's overall performance. Additionally, we calculated precision‐recall^61^ metrics because they are more informative for datasets that have a large class imbalance.^62^ The class imbalance in our dataset was caused by a high number of background pixels compared to artifact pixels. Precision (also known as positive predictive value) is the proportion of correctly detected artifacts among all artifacts identified and recall is equivalent to TPR. Like the ROC curve, we used the AUC of the precision‐recall curve to provide a metric of the overall performance. We compared the AD2 model to an analytical approach developed by Bouilhol et al.^26^. The Bouilhol method was applied to our validation dataset and the sensitivity and specificity were calculated for all images where artifacts were detected. Finally, we applied the model to the DIR‐Lab data^34^, ^35^ and visually inspected the images for model performance.

## 3 RESULTS

### 3.1 Model performance

**FIGURE 6:**
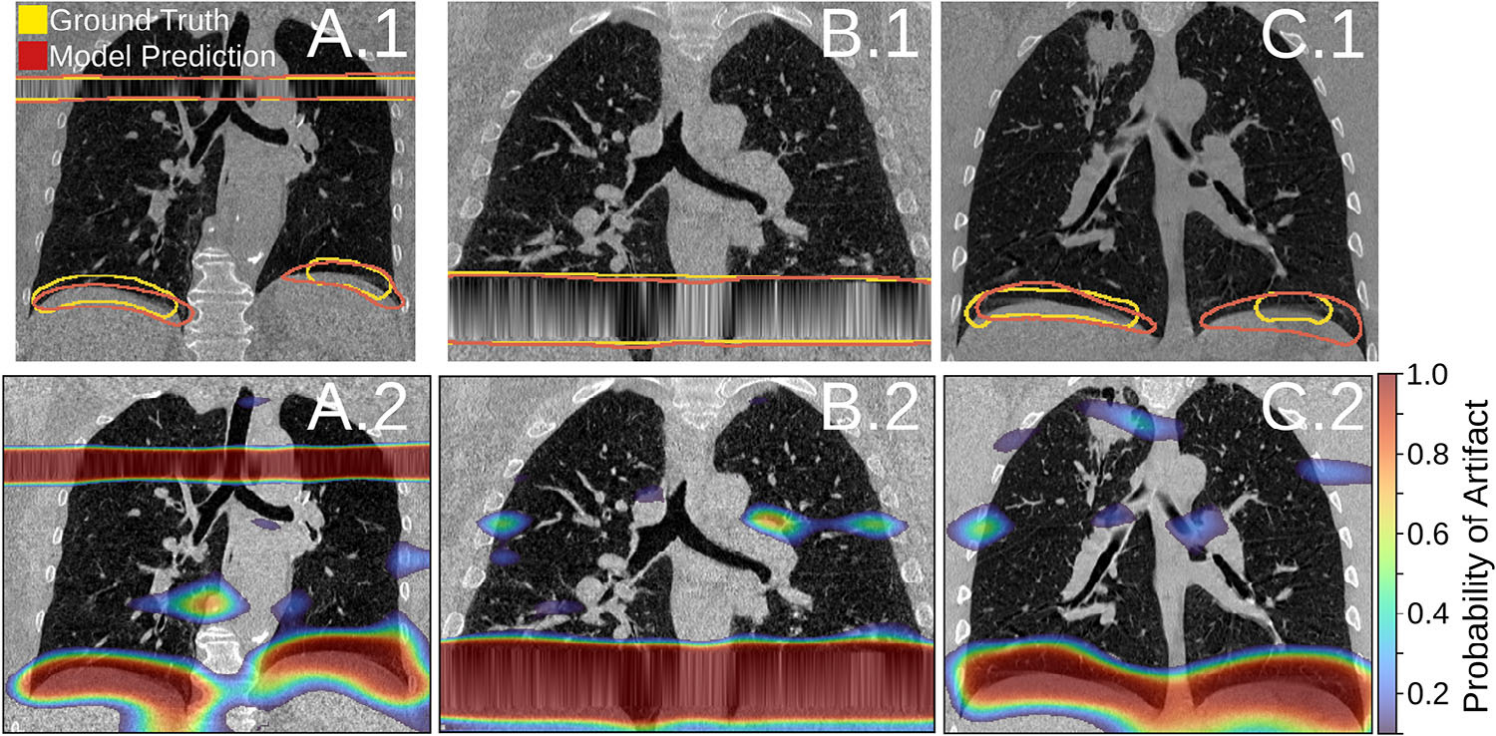
The AD2 model predicts multiple types of artifacts. Row 1 shows the model's prediction (thresholded at 0.94) compared to the human observer's segmentation. Row 2 shows the raw output of the model, a probability map, that illustrates the model's certainty of where the artifacts are located. Image A contains a small interpolation artifact at the apex and small duplication artifacts at the diaphragm. Image B contains a large interpolation artifact. Image C contains small duplication artifacts at the diaphragm.

**FIGURE 7:**
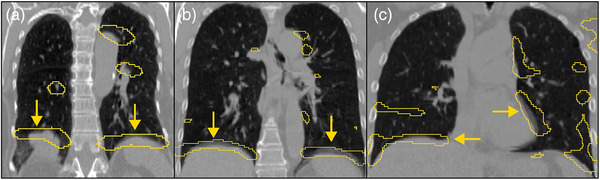
Examples of the AD2 model's performance on the DIR‐Lab Dataset.^34^, ^35^ (a) Shows two large artifacts at the diaphragm that the model was able to detect. There were some false positives located in the middle of the lung and the apex. (b) Shows a similar result to (a). Minor false positives can be seen at the apex and two large duplication artifacts at the diaphragm. (c) shows more false positives but also shows the model detecting a duplication artifact in the right lung and a misalignment artifact in the left lung.

Figure 6 gives an example model output for one of the validation images. Using the selected threshold that matches the human observer, we can clearly see that the model is able to identify several types of artifacts ranging in size and shape. In Figure 6A.1. the model segments an interpolation artifact at the apex of the lung and truncation artifacts at the diaphragm. Figure 6B.1. segments a large interpolation artifact at the diaphragm. Figure 6C.1. segments duplication artifacts at the diaphragm. Figures 6A.2., B.2., and C.2. show the raw output of the model which is a probability map. The probability map shows regions where the model predicts an artifact is present. Thresholding the probability map will yield different artifact masks and different model performance. At a probability threshold of 0.94, the model performed with a sensitivity of 0.78, specificity of 0.99, and a precision of 0.58. The Bouilhol method^26^ was evaluated on 21‐3D volumes. Among these images, 5 volumes contained a detected artifact. We calculated a sensitivity of 0.46 and a specificity of 1.00. Figure 7 shows multiple examples of the AD2 model applied to the DIR‐Lab dataset. Qualitatively, we can see that the model is able to detect some duplication artifacts and misalignment artifacts.

### 3.2 ROC and precision‐recall experiments

**FIGURE 8:**
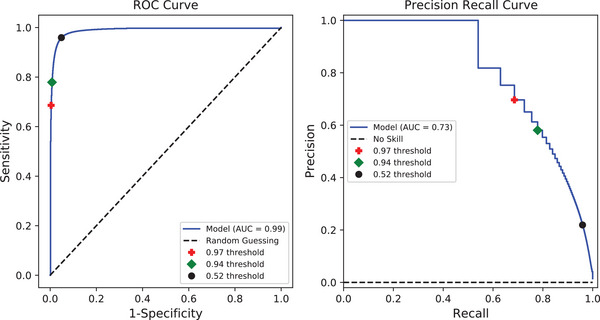
ROC and precision‐recall curves for the artifact detection model. In both cases, the model performs well achieving a good balance of sensitivity and specificity while maintaining precision in positive predictions. Depending on the threshold used, the model's performance varies. Thresholds of 0.97, 0.94, and 0.52 were plotted corresponding to the threshold determined by the F1 statistic, matching the human observer, and the Youden index respectively.

Performance of the AD2 model was evaluated using the area under the ROC curve^60^ and precision‐recall curves.^61^ On the validation dataset of 21‐3D volumes (Referenced in Section 2.3.2), the model achieved an AUC‐ROC of 0.99 and an AUC‐PR of 0.73. To compare model performance at different thresholds, we used thresholds selected by three methods: Youden's J statistic, the F1‐score, and by matching the sensitivity to the human observer. We found thresholds indicated by our metrics to be 0.52, 0.97, and 0.94 for Youden's J statistic,^60^ F1‐score,^61^ and matched the sensitivity of the human observer, respectively. In Figure 8 each threshold marker is displayed to indicate the model's performance at various points. In general, a lower threshold corresponds to a larger area of the image labeled as an artifact and an increase in false positive predictions.

### 3.3 Artifact incidence

**FIGURE 9:**
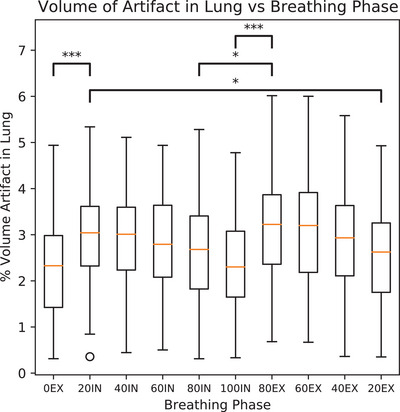
The percentage of lung labeled as artifact is shown for each breathing phase. The 80EX phase contains the largest volume of artifact in the lungs. There is statistical significance between various phases at the inflection points of inspiration and expiration. 0EX and 100IN contain the smallest volume of artifact. (* *p* < 0.05, **p < 0.01, ***p < 0.001) More details on this analysis are given in section 3.3

Figure 9 shows the average fraction of lung affected by artifact versus breathing phase as determined by the AD2 model for 79 subjects in our dataset (excluding subjects used for training). This resulted in the analysis of 790‐3D volumes. Our analysis reveals that the 0EX and 100IN phases exhibit the least volume of artifacts, while the 60EX and 80EX phases demonstrate the highest artifact levels. The distinction between expiratory and inspiratory phases shows statistical significance in cases where lung motion is rapidly changing, whereas it is not statistically significant when motion is temporally stable. Specifically, our results indicate significant differences in the amount of artifacts in the 80EX and 80IN phases (*p* = 0.012), 80EX and 100IN phases (*p* = 1.27e−5), 20EX and 20IN phases (*p* = 0.025), and 0EX and 20IN phases (*p* = 9.07e−4). Notably, among the expiratory phases, our findings highlight the significance of the 80EX phase. This phase captures the moment when the patient initiates exhaling, resulting in maximal acceleration of lung tissue. Consequently, the 80EX phase consistently demonstrates the highest volume of artifacts compared to all other examined phases.

## 4 DISCUSSION

### 4.1 Artifact detection model

It is useful to have an artifact detection tool to quantify the number of artifacts within a scan. Immediately after scanning, we can employ the artifact detection model to assess the extent of artifacts in the image and determine whether a patient re‐scan is desirable. Detecting artifacts is also a useful tool for post‐processing techniques to eliminate artifacts from the scans.

Artifacts, which notably affect 4DCT scans, present a formidable obstacle to radiation dose planning, especially when it comes to discerning true anatomy to artifact affected anatomy. While there is extensive research on the nature and origins of artifacts, establishing precise boundaries around them remains challenging due to their potential variations in shape, size, and type. Because of the wide variety of artifacts previous work^25^, ^26^, ^27^, ^29^ on artifact detection often focused on one or two types of artifacts. Here we present a deep learning artifact detection model that goes beyond detection of a single type of artifact.

Based on the ROC and precision‐recall curves, we were able to see that AD2 performed well for a variety of artifact types and sizes. While the ROC curve is good for giving an aggregate measure of model performance, it fails to describe the model in its entirety. In our dataset, which was acquired using audio guidance, artifacts make up a small percentage of the entire image. As a result, there are many true negatives in our ROC analysis. The high volume of true negatives led to a near perfect ROC curve. While the ROC curve gives a valuable visual indication of how the model's sensitivity to artifacts is consistent, it does not provide adequate information about the detailed reality of the model's performance. The precision‐recall curve provides more context on the model's performance. The model's precision gives more useful information on how AD2 segments artifacts in relation to the GT. At a threshold of 0.94, the AD2 model has a precision of 0.58, sensitivity (recall) of 0.78, and specificity of 0.99. This result is an improvement compared to previous work proposed by Bouilhol et al.^26^ On our validation dataset, their method of artifact detection performed with a sensitivity of 0.46 and specificity of 1.00. The Bouilhol method is a simple implementation for artifact detection that requires much less development than our deep learning approach. This method also did well at producing no false positives. It excelled at detecting artifacts that have a distinct discontinuity between stacks, but was unable to detect interpolation artifacts or misalignment artifacts. In their work, Bouilhol et al. write that their method was fine tuned for their institution's data which explains why their method did not perform well on our dataset.

We have demonstrated (Figure 6) that AD2 is robust to various types of artifacts and can predict multiple artifacts within a single image. Visual inspection shows that the model is able to identify most of the visible artifacts in the CT scan. Additionally, the AD2 model performs well on data collected from another institution. Figure 7 shows the model successfully identifying multiple duplication and misalignment artifacts. Although the model was not trained on the DIR‐Lab dataset, it proves it's utility and can serve as a benchmark for artifact prevalence. It's performance is not perfect as several false positives can be seen in Figure 7, however this is to be expected since the model was not optimized for that scanner and scanning parameters. Moving forward, it would be beneficial to train a model using data across multiple different scanners or to improve upon our image pre‐processing pipeline. Given that the AD2 model is able to work on another institution's data, we can see that this work could be used in a clinical setting to assess for image quality and artifact severity. In the clinic, visible artifacts are what we are most interested in as they are most impactful to a scans usability. The AD2 model proves that it can be used on many 4DCT scans to guide clinicians in searching for artifacts or be utilized as an input to a artifact correction network.

### 4.2 Clinical impact

4DCT imaging in RT is a common clinical practice sometimes limited by poor image quality. Despite pre‐processing efforts, such as phase binning, amplitude binning,^63^, ^64^ visual cues, and audible breathing cues,^15^ artifacts are still found throughout 4DCT images. Clinicians do not require respiratory motion management in approximately 30% of cases.^10^ They report that artifacts necessitate rescanning for between 5% and 14% of patients.^10^ CT image artifacts can reduce the accuracy of CT numbers (HUs) and introduce geometric distortions of the patient anatomy. Both of these errors can negatively impact RT treatment planning. A previous study of the dosimetric impact of CT image artifacts focused on those created beyond the limitations of the nominal field‐of‐view, when images are reconstructed beyond that range. Dose calculation errors caused by inaccuracy of the CT numbers were in the order of 2% –3% for 6–23 MV x‐rays.^65^ CT image artifacts can also be created by implanted high‐Z materials.^66^ A phantom study with bilateral steel inserts showed >15% dose discrepancies.^67^, ^68^ CT image artifacts caused by motion (e.g., respiratory, cardiac, peristalsis) have been long understood, and are capable of creating clinically relevant geometric errors. An object moving with an amplitude of +/−1 cm in the direction orthogonal to the imaging plane could be reconstructed with a length error of 2 cm.^69^ If left unaccounted for this would produce a geometric miss during treatment delivery. Our research group is concerned with motion artifacts that disrupt the segmentation of lung substructures (airways, vessels, fissures between lobes, etc.). With the continuous development of advanced planning techniques, efficient artifact detection is crucial to adequately evaluating scan quality. In particular, functional lung avoidance RT requires high fidelity images throughout the lung, not just in the target area. In such cases, artifacts can cause misidentification of high functioning lung regions. Recently, an alternate approach to 4DCT scanning has arisen known as sequence scanning.^70^, ^71^, ^72^, ^73^ This new technology uses adaptive learning of patient breathing patterns and dynamic scanning to produce higher quality images with minimal artifacts. Advancements in scanning protocols and new types of scanners are a viable alternative to retrospective analysis of artifact affected images.

The results presented in Figure 9 highlight several key findings from our study. Notably, the 80% expiratory phase (80EX) consistently emerged as the phase with the highest volume of artifacts within the lung. This phase, representing the transition from active (inspiration) to passive (expiration) breathing, is characterized by the expulsion of a substantial volume of air from the lung into the external environment. Moreover, Figure 9 reveals a significant difference between the 0EX and 20IN phases, signaling a marked shift in lung motion dynamics, which in turn leads to a significant increase in artifact occurrence which is likely attributed to the active nature of inhalation. A similar trend is observed between the 80EX and 100IN phases, further indicating that phases coinciding with the initiation of inspiration and expiration exhibit greater lung motion and are more likely to contain artifacts. Conversely, fewer artifacts are found in the 0EX and 100IN phases due to reduced lung movement. The statistical significance noted between the 80EX and 80IN phases underscores the variance in artifact prevalence between inspiration and expiration phases characterized by the same target lung volume. A similar distinction is observed between the 20EX and 20IN phases. We hypothesize this significant difference is due to the different mechanisms by which inspiration and expiration are performed and the large deformations the lung undergoes at these phases. Differences in artifact prevalence based on respiratory phase could provide valuable information in a clinical setting and should be investigated.

### 4.3 Limitations and future work

One limitation in our training data is that we used data acquired with audio‐guided breathing cues. This method of audio guidance is designed to reduce artifacts during the scanning process. As a result, our training dataset contained scans with an average ratio of artifact volume to lung volume between 2% and 4% as shown in Figure 9. Data acquired from free breathing patients would have resulted in a larger ratio and in more artifacts for training. However, AD2 still performed well with a limited selection of artifacts. It is important to note that some analytical methods of artifact detection are still valuable and may be faster in some regards. For example, Werner et al.^30^ demonstrated that interpolation artifacts can be detected from a simple MSD calculation. This could be further improved by applying frequency domain analysis on the resulting curve of MSD by the slice number along the *z*‐axis. This is an alternative method of performing artifact detection on interpolation artifacts that could be easily implemented.

Another limitation is the model's single class architecture. While our data contained a variety of different artifact types, the artifacts presented to the model in the training and validation data were not classified by type. It would be advantageous to create another artifact detection model that would be able to classify and distinguish the several types of artifacts. This approach would provide more information on the frequency of specific types of artifacts within a dataset and identify common causes of these artifacts. A disadvantage of using multiple models to detect each class of artifact would be increased complexity, optimization, and training time.

Moving forward, we plan to utilize our artifact detection network as an input for a deep learning artifact correction pipeline. Another area of work that would benefit the study of artifacts is the generation of artificial artifacts. To date, specific artifacts, namely duplication and interpolation artifacts, have been successfully replicated.^27^ However, there are still many artifacts that have not been artificially generated. Artificial artifacts would enable us to improve our detection model.

## 5 CONCLUSION

In conclusion, this study presented a deep learning model, AD2, for the detection of respiratory motion artifacts in 4DCT images, addressing a critical challenge in imaging lung cancer patients for RT. The impact of artifacts on the accuracy of 4DCT‐derived metrics cannot be overstated, as they often introduce significant uncertainties in RT planning and diagnostic applications.^14^, ^15^, ^17^, ^40^ Our work shows that multiple types of artifacts in 4DCT images can be effectively identified and potentially removed with the aid of our deep learning model. Artifact correction methods^27^, ^29^ can rely on AD2 to locate artifacts. While challenges and opportunities for refinement persist, the integration of AD2 into clinical practice holds great promise for advancements in RT and medical imaging. As we continue to refine and expand upon this work, we anticipate that AD2 will play a role in addressing the longstanding issue of respiratory motion artifacts.

## CONFLICT OF INTEREST STATEMENT

Joseph M. Reinhardt is a shareholder in and receives licensing fees from VIDA Diagnostics, Inc. Gary E. Christensen receives licensing fees from VIDA Diagnostics, Inc. and he is also a consultant and owns stock in PowerPollen, Inc., Ames, IA. John E. Bayouth has ownership interest in MR Guidance, LLC.
